# Feasibility of lung imaging at 0.55T for assessment of interstitial lung disease in patients with pulmonary sarcoidosis

**DOI:** 10.1007/s00330-026-12708-z

**Published:** 2026-06-27

**Authors:** Maurice Pradella, Hanns-Christian Breit, Jens Bremerich, Constantinos Anastasopoulos, Michael Bach, Oliver Bieri, Katrin E. Hostettler, Grzegorz Bauman

**Affiliations:** 1 Department of Radiology, University Hospital Basel, Basel, Switzerland; 2 University of Basel, Basel, Switzerland; 3 Department of Radiology, Division of Radiological Physics, University Hospital Basel, Basel, Switzerland; 4 Department of Biomedical Engineering, University of Basel, Allschwil, Switzerland; 5 Clinics of Respiratory Medicine, University Hospital Basel, Basel, Switzerland

**Keywords:** Low-field lung MRI, Pulmonary sarcoidosis, Ventilation–perfusion imaging

## Abstract

**Objectives:**

To assess the feasibility and diagnostic performance of radiation-free 0.55-T lung MRI for pulmonary sarcoidosis, combining morphological imaging with functional proton MRI ventilation/perfusion metrics, and to explore correlations with pulmonary function testing (PFT).

**Materials and methods:**

In this prospective study, 15 patients with pulmonary sarcoidosis and 30 healthy volunteers underwent 0.55-T lung MRI (bSTAR morphology; matrix-pencil based functional proton MRI). CT served as the reference standard for morphological findings (majority vote of experienced readers). Functional maps yielded ventilation defect percentage (VDP), perfusion defect percentage (QDP), and ventilation-perfusion overlap (VQO); thresholds were derived from the healthy cohort. Group comparisons and correlations with PFT were performed.

**Results:**

MRI achieved the highest sensitivity for consolidations (86%) and moderate performance for nodules (67%) as well as ground-glass opacities (70%), but lower sensitivity for reticulations (29%) and traction bronchiectases (27%) compared with CT. Inter-reader and inter-modality agreement were moderate. Functional MP-MRI revealed significantly higher VDP, QDP, and VQO in patients compared with volunteers (all *p* < 0.01). Strong correlations were found between MRI-derived VDP and RV/TLC ratio (*r* = 0.90, *p* < 0.001) and between QDP and FEV1 (*r* = 0.63, *p* = 0.01).

**Conclusion:**

Low-field lung MRI provides complementary information in pulmonary sarcoidosis, enabling structural assessment free of ionizing radiation, with overall moderate agreement compared to CT, and adds valuable regional lung function analysis correlated with PFT.

**Key Points:**

***Question*** *Can 0.55-T lung MRI supplement CT in pulmonary sarcoidosis by providing structural assessment plus regional ventilation and perfusion metrics linked to pulmonary function?*

***Findings*** *0.55-T MRI showed high sensitivity for consolidations (86%) and revealed increased ventilation and perfusion defects that correlated strongly with the residual volume-to-total lung capacity ratio.*

***Clinical relevance*** *Low-field lung MRI enables radiation-free assessment of structural and functional lung involvement, offering additional regional information that may support longitudinal monitoring in selected patients with pulmonary sarcoidosis.*

**Graphical Abstract:**

**Figure:**
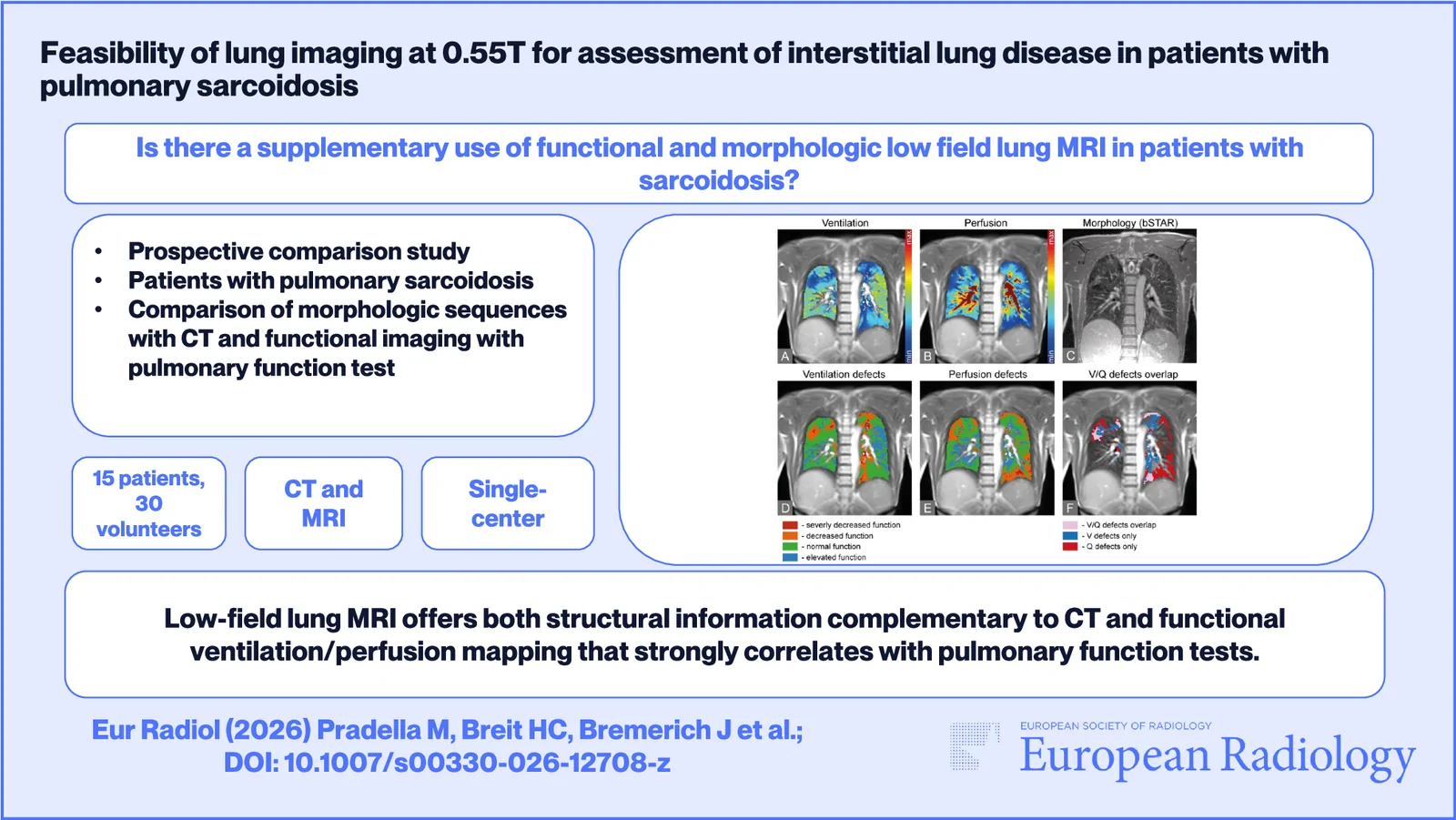

## Introduction

Sarcoidosis is an inflammatory, granulomatous disease that can manifest in various organs. Pulmonary involvement is relatively common and may lead to irreversible pulmonary fibrosis [1]. Chest CT is the imaging gold standard for assessment of pulmonary involvement but relies on ionizing radiation, a concern especially in younger patients [2]. If pulmonary involvement is excessive, functional imaging of lung perfusion or ventilation might be useful; however, this is classically the domain of nuclear medicine.

While MRI plays an essential role in diagnosing cardiac involvement in sarcoidosis [3], its clinical application for pulmonary assessment has been challenging. The combination of low proton density and strong magnetic susceptibility artifacts in the lungs leads to rapid signal decay and poor signal-to-noise ratio (SNR) with conventional methods [4]. Historically, these limitations required the use of hyperpolarized noble gases, such as ³He or ¹²⁹Xe, to generate a sufficient signal for diagnostic imaging [5, 6].

However, recent developments in hardware and pulse sequence design have enabled robust, high-quality lung imaging using native, proton MRI alone, obviating the logistical and cost challenges associated with hyperpolarized agents. Crucially, these modern techniques allow for a comprehensive examination that captures both high-resolution morphology and assessment of regional lung ventilation and perfusion [7].

Among these new proton MRI methods, techniques based on the balanced steady-state free precession (bSSFP) pulse sequences are particularly promising. bSSFP is a rapid imaging technique renowned for offering the highest SNR per unit of time among all MRI sequences [8], an advantage for lung imaging, especially at lower magnetic fields. For instance, functional techniques like Fourier decomposition MRI and matrix pencil MRI (MP-MRI) leverage bSSFP to map regional ventilation and perfusion without contrast agents [9, 10]. For high-resolution morphological imaging, a recently developed 3D half-radial dual-echo bSSFP technique, termed bSTAR, employs a minimal repetition time (TR) to generate artifact-free images of the pulmonary parenchyma and vasculature [11].

The renaissance of low-field scanners, which feature lower susceptibility-induced artifacts in the lungs, promises further opportunities [12, 13]. Magnetic field inhomogeneities are lower at a lower field strength such as 0.55 T, enabling high-resolution, large field-of-view acquisitions free of banding artifacts [14]. However, there is little experience on the clinical use of commercial 0.55-T low-field scanners in pulmonary imaging in sarcoidosis patients [15, 16].

Therefore, the aim of this study is to evaluate the potential of structural and functional lung imaging at 0.55 T in a patient population with known pulmonary sarcoidosis.

## Materials and methods

This prospective study was approved by the local ethics committee (XXXX 2021-00166). Written informed consent was obtained from each participant.

### Patient population

We enrolled 15 patients with known pulmonary sarcoidosis (mean age 50.2 ± 13.1 years, 8 females, 7 males) who underwent a routine chest CT immediately followed by research 0.55-T MRI scan. In addition, patients underwent lung function tests, which were either performed during the CT visit or at the time of MRI.

A reference cohort comprising thirty healthy, asymptomatic volunteers (mean age 33.1 ± 6.7 years; 15 females, 15 males) was prospectively enrolled to establish baseline normative values for functional lung imaging. Given the exploratory feasibility design of this study, this reference cohort was not strictly age-matched to the patient population.

### MR imaging

MRI scans were performed on a 0.55-T whole-body MR-scanner (MAGNETOM Free.Max, Siemens Healthineers) equipped with low-performance gradients (26 mT/m amplitude, 45 T/m/s slew rate). The integrated body coil was used for radiofrequency (RF) transmission and a combination of a 6-channel chest array coil, and a 6-channel spine coil was used for signal reception. Morphological lung imaging was performed by using a free-breathing 3D half-radial dual-echo bSSFP sequence termed bSTAR (4) (TA = 5:30 min, TE1/TE2/TR = 0.08/2.1/2.2 ms, field-of-view = 340 × 340 × 340 mm^3^, flip angle = 25°, bandwidth = 1002 Hz/pixel) with a 1.3 mm isotropic resolution.

Furthermore, functional lung MP-MRI data were acquired in free-breathing using two-dimensional time-resolved ultra-fast bSSFP (13) (TE/TR = 1.1/2.7 ms, TA per image = 202 ms, TA per slice = 45 s, field-of-view = 475 × 475 mm^2^, in-plane resolution = 3.7 × 3.7 mm^2^, slice thickness = 14 mm, flip angle = 70°, bandwidth = 908 Hz/px, 160 coronal images per slice). To cover the whole lung, 8–12 slices were acquired, which resulted in an overall scanning time of 6–10 min.

### CT imaging

Chest CT scans were acquired in supine position using either a 128-slice SOMATOM Definition Edge or a 2 × 192-slice SOMATOM Force (Siemens Healthineers) scanner. Mean tube voltage was 105.0 kVp (standard deviation (SD): 10.1), mean tube current-time product 81.1 mAs (SD: 19.2), and pitch factor 1.05 in all cases. All scans were performed without contrast. Images were reconstructed in 1-mm slice thickness using lung and soft tissue reconstruction kernels.

### Image evaluation

Three subspecialized cardiothoracic radiologists (experience: 20 years, 6 years, 1 year) independently reviewed first the CT scans and, after a blinding period of minimum 7 days, the MRI scans of each patient. Raters were asked to evaluate the presence of reticulations (RET), ground-glass opacities (GGO), traction bronchiectases (TB), consolidations (CON), and nodules (NOD) for the respective imaging study. Additional findings such as honeycombing, thickened interlobular septa, pleural thickening, and pleural effusions were reported separately, if present.

Interrater agreement was quantified using Fleiss kappa (K) for the CT and MRI reading sessions. Inter-modality agreement (CT vs. MRI) per rater was assessed with Cohen’s kappa. The majority vote of 2 readers in the CT was defined as the gold standard. Based on this, sensitivity and specificity were calculated for RET, GGO, TB, CON, and NOD in the MRI.

### MP-MRI data evaluation

The color-coded maps of pulmonary ventilation and perfusion were automatically generated from the ultra-fast bSSFP datasets using a dedicated image analysis software—TrueLung [17]. The signal distributions on the automatically segmented ventilation and perfusion maps obtained in healthy volunteers were used to estimate threshold values indicating functional impairment in sarcoidosis patients [18]. Instead of employing a median threshold as in previous studies with MP-MRI [19, 20], we used a binning method to quantify pulmonary function. Similar approaches were previously used to quantify ventilation using hyperpolarized gas lung MRI. The proposed method employs an exponentially modified Gaussian (EMG) distribution [21] to model the ventilation and perfusion signal. The probability density function is expressed as follows:

$${{\rm{pdf}}}\left(x\right)=\,\frac{\lambda }{2}{e}^{\frac{\lambda }{2}\left(2\mu -\lambda {\sigma }^{2}-2x\right)}{{\rm{erfc}}}\left(\frac{\mu +\lambda {\sigma }^{2}+x}{\sqrt{2}{\sigma }^{2}}\right)$$

where λ is the exponential rate, μ the mean, and σ^2^ the variance of the Gaussian component, erfc is the complementary error function. The mean of the EMG is given by ${\mu }_{{EMG}}=\mu +1/\lambda$ and the variance by ${\sigma }_{{EMG}}^{2}={\sigma }^{2}+1/{\lambda }^{2}$. Based on the EMG distribution fitted to mean normalized data from all healthy volunteer, we defined four bins corresponding to severely decreased function (SDF) [$0,\,{\mu }_{{EMG}}-2{\sigma }_{{EMG}}$], decreased function (DF) [${\mu }_{{EMG}}-{2\sigma }_{{EMG},}{\mu }_{{EMG}}-{\sigma }_{{EMG}}$], normal function (NF) [${\mu }_{{EMG}}-{\sigma }_{{EMG},}{\mu }_{{EMG}}+{\sigma }_{{EMG}}$] and elevated function (EF) [${\mu }_{{EMG}}+{\sigma }_{{EMG},}\infty$].

Global outcomes per subject were ventilation defect percentage (VDP), perfusion defect percentage (QDP), and ventilation/perfusion defect overlap (VQO). VDP and QDP were defined as the percentage of the area classified either as SDF or DF to the total segmentation volume in each subject. Similarly, VQO was calculated as the percentage of the area of overlapping ventilation and perfusion defects to the total segmentation volume. Correlations between functional indices from MRI and conventional lung function tests were calculated with Spearman’s correlation coefficients. Due to the limited sample size and non-normal distribution of the functional MP-MRI data, nonparametric statistical tests were utilized. Group comparisons of continuous variables (VDP, QDP, and VQO) between sarcoidosis patients and healthy volunteers were performed using the Mann–Whitney U test. Correlations between MRI-derived functional indices and pulmonary function tests were evaluated using Spearman’s rank correlation coefficient. To address the issue of multiple comparisons in the correlation analysis between MRI-derived metrics and pulmonary function tests, the false discovery rate (FDR) was controlled using the Benjamini–Hochberg procedure [22]. An FDR of 5% (q = 0.05) was defined as the significance threshold.

## Results

Demographics of patients and healthy volunteers are displayed in Table 1. MRI and CT examinations were successfully completed without any adverse effects in all 15 patients. Median time between CT and MRI was 41 days [IQR 21; 68]. Functional MRI sequences were also successfully performed in all 30 volunteers. All image series were fully acquired and reconstructed without any data loss.

**Table 1:** Demographics of patients and healthy volunteers

Parameter	Sarcoidosis patients	Health volunteers
Number of subjects	15	30
Age (years)	50.2 ± 13.1	33.1 ± 6.7
Females, males	8, 7	15, 15
Height (cm)	168.5 [158.0, 174.5]	176.7 [170.2, 185.0]
Weight (kg)	71.0 ± 16.3	72.7 ± 11.8
BMI (kg/m^2^)	25.0 ± 5.7	23.1 ± 2.0

Values are either reported as numbers or, depending on the parameter distribution, as mean ± standard deviation (normal distribution) or median [interquartile range] (non-normal distribution)

*BMI* body mass index

### Image-based evaluation

Ground glass was seen by at least 2 of 3 readers in 10 of 15 patients in MRI and in 8 of 15 patients in CT. Nodules were seen in 8 of 15 patients in MRI and in 14 of 15 patients in CT. Tractions bronchiectases were seen in 1 of 15 patients in MRI and in 4 of 15 patients in CT. Reticulations were seen in 0 of 15 patients in MRI and in 7 of 15 patients in CT.

**Fig. 1:**
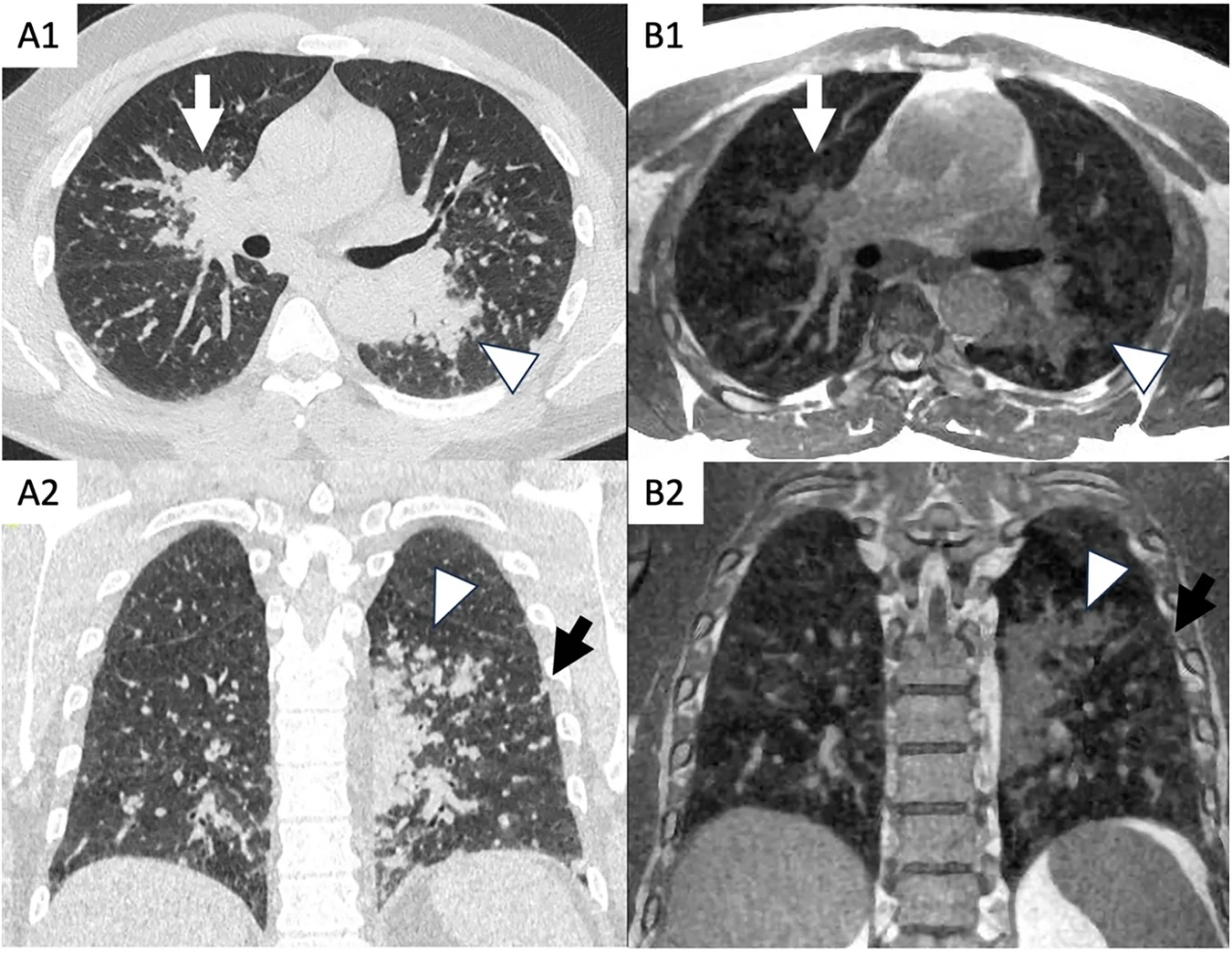
Transversal and coronal CT (**A1**, **A2**) and MRI (**B1**, **B2**) images of a 38-year-old male patient with pulmonary sarcoidosis. Consolidations are visible in the right upper lobe (white arrows) and left lower lobes (white arrowheads) on both CT and MRI images. Single nodules are harder to detect on MRI images (black arrows)

**Fig. 2:**
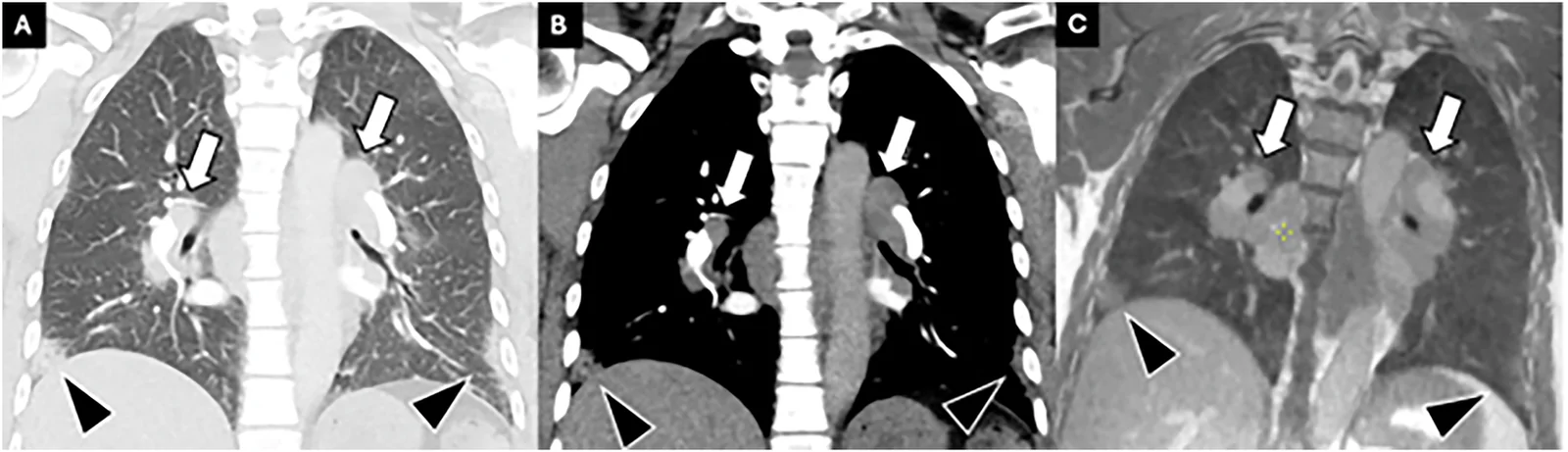
Coronal CT (**A** lung kernel, **B** soft tissue kernel) and MRI (**C**) images of a 52-year-old female patient with pulmonary sarcoidosis. Consolidations are visible in the basolateral lower lobes on both sides (black arrowheads) on both CT and MRI images. Bihilar lymphadenopathy is also visible (white arrows)

Pleural thickening was seen in 0 of 15 patients in MRI and in 1 of 15 patients in CT. Mediastinal lymphadenopathy was seen in 10 of 15 patients in MRI and in 14 of 15 patients in CT. Honeycombing and pleural effusion were seen in 0 of 15 patients in both MRI and CT. Image findings of two exemplary patients on MRI and CT are exemplarily shown in Figs. 1 and 2.

Inter-reader agreement was moderate (R1-2: 0.567, CI: 0.508–0.621; R1-3: 0.454, CI: 0.395–0.513; R2-3: 0.441, CI: 0.385–0.497). Inter-modality agreement was also moderate for all readers (R1: 0.440, CI: 0.380–0.456; R2: 0.336, CI: 0.282–0.392; R3: 0.481, CI: 0.435–0.533).

Mean accuracy, sensitivity and specificity between CT and MRI readings amounted to (accuracy, sensitivity, specificity): consolidations (73%, 86%, 66%), traction bronchiectases (73%, 27%, 96%), nodules (71%, 67%, 67%), ground-glass opacities (64%, 70%, 59%), and reticulations (69%, 29%, 86%).

### MP-MRI data evaluation

**Fig. 3:**
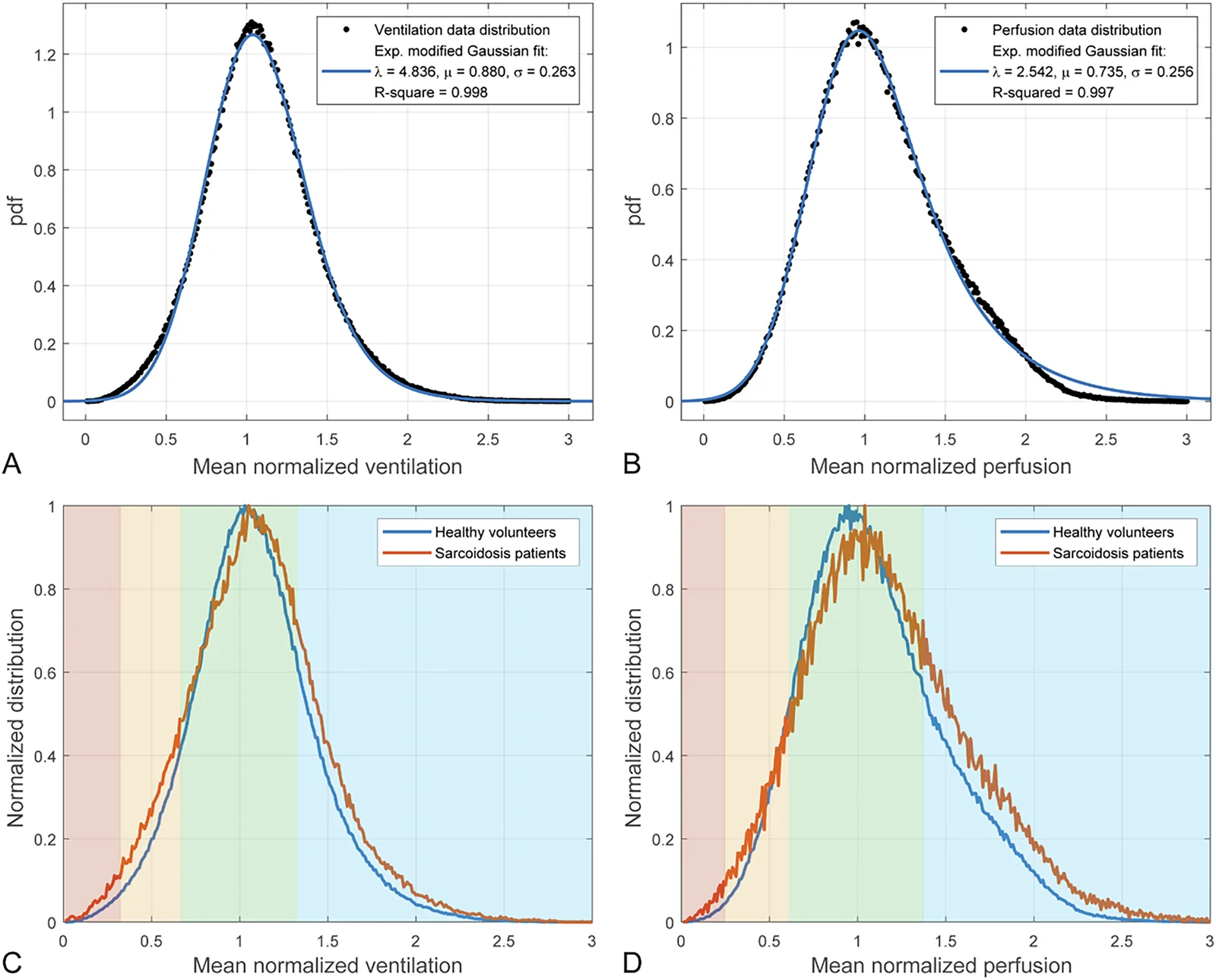
Exponentially modified Gaussian distributions fitted to mean normalized distributions of ventilation (**A**) and perfusion (**B**) measured in all datasets from healthy volunteers. Diagrams in the bottom row (**C** and **D**) show signal intensity bins defined as: SDF—severely decreased function (red), DF—decreased function (orange), NF—normal function (green), and EF—elevated function (blue). The bins are overlaid on mean normalized distributions from sarcoidosis patients and healthy volunteers

**Fig. 4:**
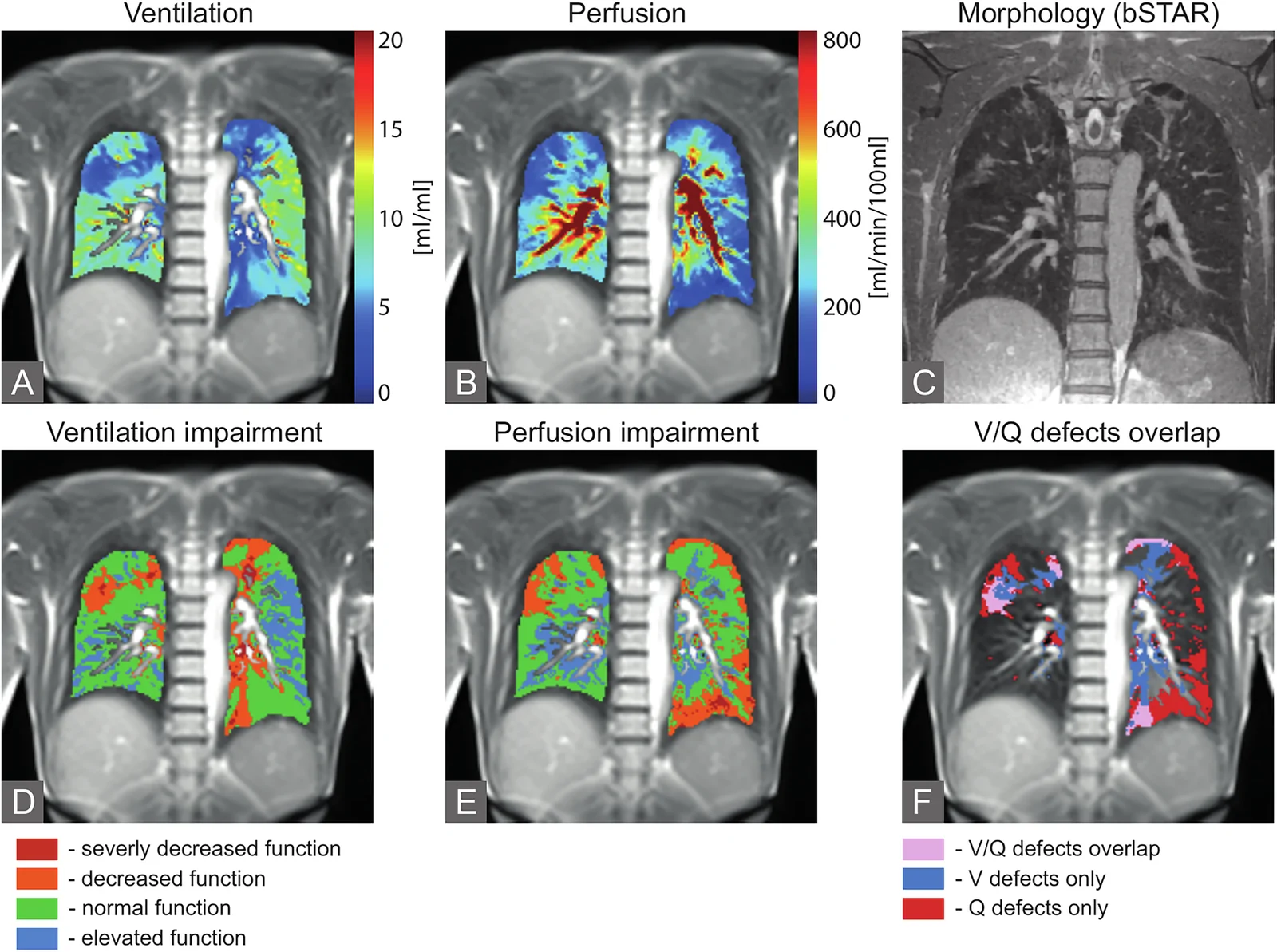
Exemplary images obtained in a 46-year-old female sarcoidosis patient showing ventilation (**A**), perfusion (**B**), morphological bSTAR^8^ acquisition (**C**), ventilation and perfusion defect maps visualized using the binning method based on the exponentially modified Gaussian model (**D**, **E**), and a map showing the overlap between ventilation and perfusion defects (**F**)

**Fig. 5:**
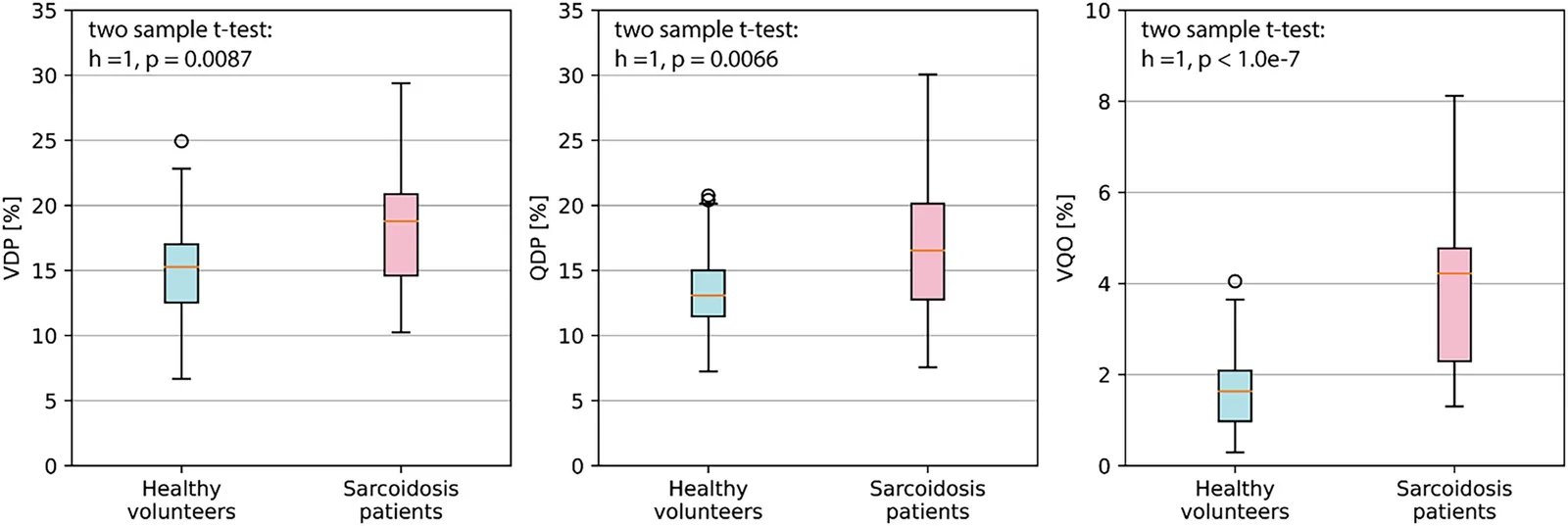
Distributions of the ventilation defect percentage (VDP), the perfusion defect percentage (QDP), and the ventilation/perfusion defect overlap percentage (VQO) calculated for healthy subjects and sarcoidosis patients. The distributions were compared using the nonparametric Mann–Whitney U test

**Fig. 6:**
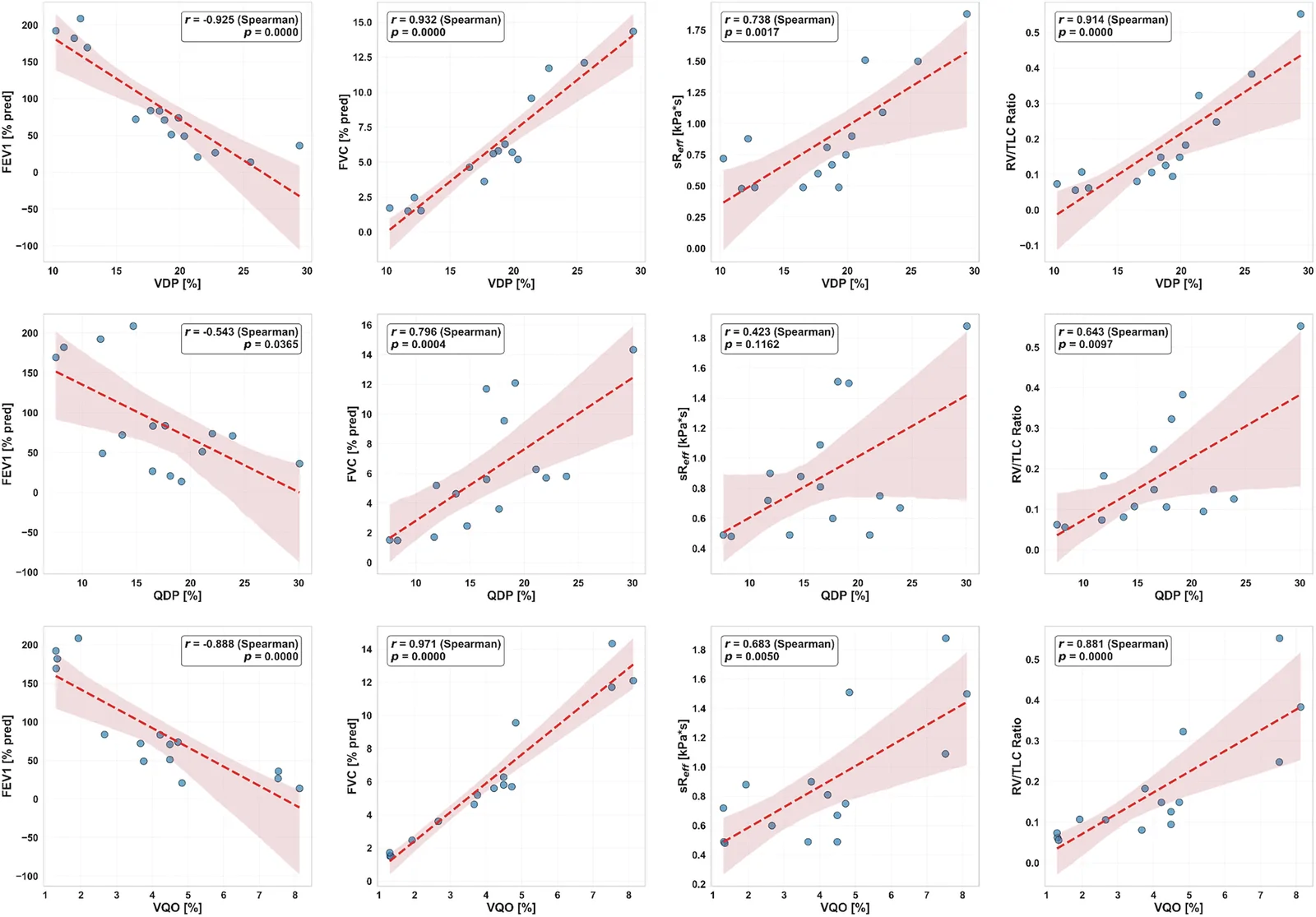
Diagrams present plots of the ventilation defect percentage (VDP), the perfusion defect percentage (QDP), and ventilation/perfusion defect overlap (VQO) measured using MP-MRI in sarcoidosis patients compared to outcomes of lung function tests, as well as Spearman correlations. FEV1, forced expiratory volume in 1 s; FVC, forced vital capacity; RV, residual volume; sR_eff_, specific effective airway resistance; TLC, total lung capacity

Median time between PFT and MRI was 17.5 days [IQR 10.5; 28.5]. Figure 3 shows EMG distributions fitted to the mean normalized distributions of ventilation (Fig. 3A) and perfusion (Fig. 3B) from healthy subjects, as well as the estimated bins overlaid on signal distributions from sarcoidosis patients and healthy subjects. Exemplary MP-MRI maps obtained in a sarcoidosis patient are presented in Fig. 4. VDP, QDP, and VQO distributions calculated for all healthy subjects and sarcoidosis patients are visualized in Fig. 5. After controlling the false discovery rate at 5%, 10 of the 15 correlations remained statistically significant. The best correlation between conventional lung function and MRI parameters in sarcoidosis patients was found for the RV/TLC ratio (vs. VDP: *r* = 0.9, *p* < 0.001, vs. QDP: *r* = 0.75, *p* = 0.01) and FEV1 (vs. VDP: *r* = 0.84, *p* < 0.001, vs. QDP: *r* = 0.63, *p* = 0.01). All other correlations can be found in Fig. 6 and Table 2. VDP was significantly lower in healthy volunteers (median, 0.153; IQR, 0.126–0.170) than in patients (median, 0.188; IQR, 0.146–0.209; *p* = 0.021). Similarly, QDP was significantly lower in healthy volunteers (median, 0.131; IQR, 0.115–0.150) than in patients (median, 0.165; IQR, 0.128–0.201; *p* = 0.034). VQO was also significantly lower in healthy volunteers (median, 0.016; IQR, 0.010–0.021) than in patients (median, 0.042; IQR, 0.023–0.048; *p* < 0.001).

**Table 2:** Outcomes of MP-MRI versus conventional lung function tests in the 15 patients with sarcoidosis

	VDP		QDP		VQO	
	r	*p*-value	r	*p*-value	r	*p*-value
FEV_1_ (%)	−0.836	0.0001*	−0.625	0.0149*	−0.794	0.0004*
FVC (%)	−0.736	0.0025*	−0.457	0.0888	−0.722	0.0024*
RV/TLC (%)	0.900	< 0.0001*	0.750	0.0129*	0.863	< 0.0001*
sR_eff_ (kPa·s)	0.738	0.0017*	0.423	0.1162	0.683	0.0050*
DLCO (%)	−0.3107	0.2592	−0.275	0.3203	−0.168	0.5493

Data are presented as Spearman correlation coefficients and *p*-values. Asterisk (*) denotes correlations that remained significant after applying the Benjamini–Hochberg procedure to control for a false discovery rate of 5%

*DLCO* diffusing capacity of the lungs for carbon monoxide, *FEV*_*1*_ forced expiratory volume in 1 s, *FVC* forced vital capacity, *RV/TLC* residual volume/total lung capacity, *sR*_*eff*_ specific effective airway resistance, *VDP* ventilation defect percentage, *VQO* ventilation-perfusion defect overlap, *QDP* perfusion defect percentage

## Discussion

The aim of this study was to evaluate the potential of structural and functional lung imaging at 0.55 T in a patient population with known pulmonary sarcoidosis. The structural assessment using bSTAR at low-field MRI demonstrated moderate agreement between readers and compared to CT, which served as the gold standard. MRI showed the highest sensitivity for detecting consolidations (86%), with moderate performance for nodules and ground-glass opacities but was less sensitive for fine structures such as reticulations and traction bronchiectases. These limitations can be explained by inherent challenges of lung MRI, including lower spatial resolution compared to CT, reduced signal-to-noise ratio at low field strength, and the susceptibility of MRI to motion-related artifacts. In particular, the detection of fine interstitial changes such as reticulations remains challenging due to the limited depiction of subtle parenchymal structures. Our findings align with those of Chung et al, who also reported that proton MRI is less sensitive than CT for detecting fine interstitial changes like reticulations and micronodules in sarcoidosis patients [23]. The sensitivity and specificity for MRI to detect pulmonary changes are, compared to other studies, partly lower. For example, Gorkem et al reported higher values with a sensitivity of over 80%. It should be noted, however, that their study was conducted on only seven patients [24]. Regarding nodule detection compared to CT, Ohno et al reported equivalence using a 3-T scanner, while Hinsen et al reported higher accuracy at 0.55 T, which depended on nodule size [25, 26]. Brady et al demonstrated that late gadolinium-enhanced MRI reliably detected and quantified pulmonary fibrosis in 16 sarcoidosis patients, showing strong correlation with HRCT findings [27]. Although their use of gadolinium-based contrast agents to assess lung fibrosis differs from our non-contrast-enhanced approach, it highlights the versatility of MRI in probing different pathophysiological aspects of the disease. However, the scanner used, the patient cohort, and the sequence technique are not comparable to our study. In particular, the use of a free-breathing, high-resolution (1.3 mm isotropic) sequence appears promising and provides a basis for further studies in cohorts such as patients with interstitial lung disease or thoracic malignancies [14, 16]. In a recent study, the bSTAR sequence demonstrated the feasibility of submillimeter morphologic imaging when applied on a low-field MR-scanner equipped with a high-performance gradient system [14]. Our work confirms the robustness of this sequence even on a commercial low-field scanner with low-performance gradients, which is a crucial step for wider clinical adoption.

The functional MP-MRI analysis revealed impairments in ventilation and perfusion in sarcoidosis patients, with moderate to strong correlations to standard pulmonary function test parameters. This suggests that low-field MRI can additionally provide valuable functional information and may complement traditional imaging and pulmonary function tests, especially in identifying regional ventilation-perfusion mismatches. This is in line with current literature: On the one hand, several studies have already shown that the severity of pulmonary sarcoidosis correlates with functional parameters [28]. On the other hand, MR-based regional quantification of ventilation and perfusion has also been demonstrated in several studies [29, 30]. To our knowledge, this study is the first to show this in a larger cohort of sarcoidosis patients.

However, functional imaging of the lung is not exclusive to MRI. Recent advances in CT technology, including dual-energy CT, photon-counting CT, and CT-based functional imaging approaches such as pulmonary blood volume mapping and ventilation-perfusion (V/Q) imaging, enable high-resolution structural and functional assessment within a single short acquisition [31–34]. These techniques offer excellent spatial resolution and increasingly allow for functional characterization of lung disease in clinical routine. In this context, the additional value of MRI should be interpreted in a complementary manner, particularly in scenarios where radiation-free imaging and repeated follow-up examinations are desirable.

Our study has several limitations that need to be addressed: First, the patient cohort is relatively small. However, this is due to the low incidence of sarcoidosis and, in particular, pulmonary fibrosis. Most studies that have systematically evaluated the performance of pulmonary MRI in sarcoidosis are based on smaller cohorts. Furthermore, this study represents a single-center, single-vendor exploratory feasibility study, which may limit the generalizability of the findings to other institutions, scanner types, and technical implementations. In addition, due to the limited sample size, age-adjusted analyses were not feasible, and potential age-related effects cannot be excluded. The diagnostic performance of MRI for detecting nodules, ground-glass opacities, traction bronchiectases, and reticulations remains inferior to CT. This might be explained by the fact that pulmonary changes in sarcoidosis are often relatively small and therefore require high(er) resolution. Notably, lesion burden was not quantitatively assessed. The higher detection rate of nodules and reticulations on CT is likely related to its superior sensitivity for small lesions, whereas MRI predominantly depicts larger or more confluent abnormalities. The median time between CT and MRI or PFT and MRI was about 40 days, which might impair comparability between methods. This was mainly due to the availability of the MRI scanner as well as patient referrals. However, no major short-term temporal changes are anticipated in the pulmonary involvement of sarcoidosis. Additionally, while we found moderate to strong correlations between conventional and MRI-based lung functional parameters, some relationships (e.g., FEV1 and QDP) should be interpreted as associative rather than causal. The reference cohort for functional imaging consisted of only 30 healthy volunteers. To establish age-adjusted normative values, larger and stratified cohorts are needed. The performance of the low-field scanner should have ideally been compared to that of 1.5-T or 3-T scanners to analyze effects caused by field strength or gradient systems. While low-field MRI at 0.55 T may offer potential advantages such as reduced susceptibility effects in lung imaging, improved patient comfort (e.g., due to wider bore systems), and possible economic and ecological benefits, these must be weighed against relevant limitations. In particular, lower gradient performance and the currently limited availability of low-field systems may affect spatial resolution and restrict broader clinical implementation. However, this was not feasible due to logistical reasons and should be addressed in future studies.

Overall, our data suggest that pulmonary imaging to assess morphologic changes with low-field MRI is still inferior to CT. However, our exploratory study indicates that a key advantage of lung MRI lies in combining structural with additional functional imaging capabilities. It enables a complementary assessment of lung perfusion and ventilation. Compared to conventional pulmonary function testing, this approach provides spatial information about the location of potential perfusion and/or ventilation defects. Therefore, lung MRI may be a valuable option for longitudinal follow-up and therapy monitoring in selected patient populations, particularly in scenarios where radiation-free imaging is desirable, rather than as a replacement for CT in routine clinical assessment.

## Data Availability

The data underlying this article will be shared on reasonable request to the corresponding author.

## Abbreviations

bSSFP: Balanced steady-state free precession
bSTAR: Half-radial dual-echo balanced steady-state free precession sequence
FEV1: Forced expiratory volume in 1 s
FVC: Forced vital capacity
MP: Matrix pencil
PFT: Pulmonary function test
QDP: Perfusion defect percentage
RV/TLC: Residual volume/total lung capacity
VDP: Ventilation defect percentage
VQO: Ventilation and perfusion defect overlap
